# Analysis of Chip Morphology in Heavy Milling of 508III Steel Considering Different Tool Wear Conditions

**DOI:** 10.3390/ma17163948

**Published:** 2024-08-08

**Authors:** Rui Guan, Yaonan Cheng, Jing Xue, Shilong Zhou, Xingwei Zhou, Wenjie Zhai

**Affiliations:** 1 Key Laboratory of Advanced Manufacturing Intelligent Technology, Ministry of Education, Harbin University of Science and Technology, Harbin 150080, China; guairui1990@163.com (R.G.); xjing0222@163.com (J.X.); zhoushilong0580@163.com (S.Z.); zxingwei2023@163.com (X.Z.); 18646133492@163.com (W.Z.); 2 Industrial Center, Harbin Vocational and Technical University, Harbin 150081, China

**Keywords:** heavy milling, 508III steel, chip morphology, chip free surface, chip curling

## Abstract

During the process of chip formation, the chip is subjected to extrusion pressure, friction, heat, and a strong chemical reaction. The chip’s macro and micro morphology, to a certain extent, reflect the condition of the tool during the cutting procedure. Therefore, researching the macroscopic and microscopic morphology of the chip’s surface in response to different tool wear conditions is of great significance to reproducing the cutting condition and analyzing the tool wear mechanism. This paper focuses on the chips formed by milling the difficult-to-machine material 508III high-strength steel. Firstly, the 508III steel milling experiment is carried out at the actual machining site to collect chip data under different tool wear conditions. Next, the free surface morphology of chips and the bottom surface morphology of chips are analyzed. Further, the chip edges are investigated, and their causes are analyzed. Finally, heavy milling 508III steel chip curl morphology analysis is performed. The research results play important roles in revealing the mechanism of tool wear and the relationship between chip morphology and tool wear. This information can be used to provide theoretical and technical support for monitoring the tool wear status based on chip morphology.

## 1. Introduction

The phenomenon of tool wear in the heavy milling water chamber head has been a key focus of scholarly investigation. This issue is particularly prominent since the 508III steel material has high strength and hardness, resulting in substantial tool wear during the milling process. The inability to detect tool wear during cutting operations can have detrimental effects on the safety of the equipment itself and that of the people who use it, as well as on regular production activities. This has a significant impact on the efficiency and precision of milling operations. Therefore, the identification and continuous monitoring of tool wear are essential to ensure the safety of equipment and personnel, as well as the uninterrupted flow of production processes [1,2]. Chips are the byproducts created when a tool cuts away excess metal layers. The cutting process generates significant heat and force, which influence the morphology of any tool that comes into direct contact with the chips. The morphology of the chips accurately indicates the tool’s wear condition. Many scholars have conducted relevant research on chip morphology and its relationship with tool wear, and their work provides a specific reference for the research in this paper.

The essence of cutting processing is the process of cutting off the excess metal layer with the tool, forming chips and the machined surface. In the process of tool cutting and machining, the workpiece material undergoes intense plastic deformation in the cutting region, generating shear-slip action and flowing over the cutting tool’s front face, which undergoes intense contact and friction to form the chip [3]. There is a significant amount of friction between the cutting tools, chips, and workpieces during milling, resulting in the generation of a considerable amount of thermal energy. The contact and friction between the chip and the tool’s cutting edge transfer the heat produced during milling. This heat-transfer mechanism plays a crucial role in determining the cutting temperature of the tool. High cutting temperatures can reduce cutting efficiency and have adverse effects on the tool’s lifespan [4,5]. Tool wear is a fundamental phenomenon in cutting processes, where the cutting edge of the tool gradually loses its sharpness, shape, and size due to various factors such as friction, heat, and vibration during the cutting process. The study conducted by the author’s research team aimed to investigate the tool wear mechanism of milling water chamber head material. The study’s conclusions showed that the main wear mechanisms impacting the cemented carbide tool were adhesive and abrasive wear when the cutting temperature was kept below 600 °C. However, once the temperature surpassed 600 °C, the dominant wear mechanism shifted to adhesive and diffusive wear, with some degree of abrasive wear still evident [6,7]. Therefore, the tool wear mechanism is directly influenced by the cutting temperature. The cutting temperature is predominantly generated by the intense friction between the tool and the chip, as well as the heat transfer that occurs during their interaction. The chip and the tool have greater contact and friction when the temperature rises, which wears down the tool’s front face. Consequently, this wear alters the rake angle of the tool, thereby impacting the macroscopic and microscopic morphology of the chip.

Wu et al. [8] established a chip curl radius prediction model and chip breakage model in high-temperature alloy cutting under high-pressure cooling to study the variation of the chip curl radius under different coolant pressures. Based on the geometric relationship and cutting parameters in the velocity vector end diagram, the difference in flow velocity between the bottom and top layers of the chip was used to obtain the chip curl radius. Devotta et al. [9] used computed tomography (CT), high-speed imaging, and Kharkevich model equations to determine a chip morphology characterization method, which can evaluate the chip morphology prediction accuracy of FE models. A three-dimensional finite element model was utilized to simulate the chip formation process of AISI 1045 steel cutting tools at different feed rates and cutting depths. Saraf et al. [10] used slotless inserts and WC microstructured inserts to turn TiAl4V under dry, vacuum, micro-lubricated, and humid conditions. The texture pattern of the inserts reduced the chip–tool contact area by promoting tighter curling of the chips. It was also found that under compressed air conditions, the chip curling radius was reduced by up to 52.3%. Sun et al. [11] researched the impact of tool wear on chips during dry turning of Ti-6Al-4V. The study revealed that the geometric variables of chips change with the increase in the material volume removed, as well as with the changes in tool geometry and cutting temperature. Wagner et al. [12] explored the chip formation, cutting process, and tool wear evolution when cutting the titanium alloy Ti-5553. Stress, temperature, and friction within the workpiece material and at the tool–chip interface are quantified. Furthermore, the chip’s micromorphology is observed. Further, morphological parameters are explored, e.g., the thickness of the shear zone or the frequency of occurrence of primary shear zones. Cui et al. [13] studied the effects of cutting force, tool temperature, tool stress, and cutting wear in experimental experiments and finite element simulations of ultra-high-speed (*v* = 200–2000 m/min) milling. The results indicate that as the cutting speed increases, the degree of chip serration becomes more and more pronounced. Segmentation is a frequently observed phenomenon that occurs in the majority of sawtooth chips at a cutting speed of 2000 m/min. The increased temperature within the shear band significantly influences the formation of cracks within the chips, leading to their separation into distinct segments. To determine the reasonable cutting parameters of AISI 52100 steel under a dry environment and minimal quantity lubrication (MQL) conditions, Havva et al. [14] characterized tool wear using scanning electron microscopy and analyzed the macroscopic morphology of chips to determine the impact of cutting conditions. Gdula et al. [15] investigated the tool life, wear forms and mechanisms, chip and machining surface morphology, and multi-axis cutting kinematics changes of nickel based high-temperature alloy ring milling cutters with circular inserts during multi-axis milling. It was found that tool wear began with steady wear and debris near the cutting depth and then developed into back-face wear through abrasive and adhesive wear mechanisms, ultimately forming notches and peeling. Das et al. [16] conducted a study to evaluate the machinability of lead-free brass in high-speed dimensional turning through an analysis of surface morphology, chip edge formation, chip formation, and tool wear. The researchers noted that chips exhibited serrations along their edges at lower cutting speeds, with a decrease in serration intensity as the cutting speed rose. Moreover, at a lower cutting speed, chips demonstrated a spiral or helical configuration, while at a higher cutting speed, chip morphology evolved into a blend of spiral, arc, and strip shapes. Che et al. [17] employed a temperature–displacement coupled penetrating contact algorithm to construct a thermo-mechanical coupled three-dimensional numerical model of the tool. This model revealed the mechanism of chip formation and the spatial distribution patterns of the elevated temperature zone.

Chip morphology and color are important indicators for in-depth revelations about heavy milling mechanisms, as well as for evaluating tool wear conditions. Most of the existing studies focus on the impacts of different cutting parameters and cooling conditions on chip formation, and it is more common to analyze and characterize sawtooth-shaped chips. It usually takes the corresponding changes in chip and tool wear as a measure to evaluate the effectiveness of new methods or to determine the reasonable cutting parameters, but seldom involves the characterization of banded chips under time-varying tool wear. The authors of this paper consider the marked variations in chip color under different tool wear conditions and utilize chip spectroscopy as a sensitive feature that characterizes chip color to identify and classify tool wear states, thereby laying a solid foundation for our current research endeavors [18].

Under heavy milling conditions, due to the large cutting parameters, the chip characteristics and morphology are different from those of conventional chips, and the cutting mechanism changes. With the intensification of tool wear and the emergence of groove wear, the chip’s macro- and micro-morphology have changed. The chip curl form and radius, chip color and chip free surface, bottom surface, and edges characteristics have changed. The formation of chips is the result of the instantaneous action of the tool and the workpiece. When tool wear is generated, and the degree of action of the workpiece is more and more intense, there is a resulting rise in the cutting temperature. It also makes the degree of cutting deformation change. Meanwhile, the ontological relationship of the material also changes. An investigation is conducted on the chip morphology of heavy milling 508III steel under various tool wear conditions to reveal the relationship between chip morphology and tool wear. The aim of this research was to elucidate the failure mechanism of heavy milling tools by analyzing chip morphology and tool wear, thereby establishing a theoretical basis and offering technical support. The contributions of this study are as follows:

(1) Investigating the macroscopic and microscopic changes in chip morphology under different tool wear conditions lays a theoretical foundation for identifying tool wear and breakage states based on chip morphology.
(2) The scratching and plowing phenomena observed on the chip’s bottom surface reflect the severity of tool–chip friction, which to some extent characterizes the tool wear state.
(3) Comparing heavy milling conditions with conventional cutting conditions allows us to explore the significant differences in the chip edge generated at the non-free and free ends of chips.
(4) The study reveals the reasons for the differences in the microscopic morphology of the chip’s free surface under different tool wear conditions.

## 2. Heavy Milling 508III Steel Experiments

### 2.1. Workpiece Material Characteristics

The research focuses on a specialized material intended for use in the water-chamber head of a nuclear power plant located within a nuclear island. This material, known as low-carbon alloy steel, is renowned for its outstanding performance. The addition of ferrophilic iron groups elements such as Mn, Ni, and Mo to 508III steel enhances its weldability, hardening capacity, and resistance to neutron irradiation embrittlement, thereby improving its strength properties. Table 1 lists the essential chemical composition in detail. Table 2 shows the mechanical properties of 508 III steel.

### 2.2. Experimental Program

The experiment is conducted in a heavy industry to determine the real chip pattern and tool wear when cutting 508III high-strength steel. The machine tool is a CNC-modified 160 boring machine; the cutter plate model number is AHFC160R11-19 0092929328. Three inserts are mounted symmetrically on the circumference of the milling cutter plate, and the inserts are TiAlTaN-coated tungsten carbide inserts; the model number is XOLX 190615SR-M50 CTPP235 of Ceratizit in Breitenwang, Austria. The primary reasons for selecting three cutting inserts are as follows: (1) In the experiment, three inserts are installed primarily to accelerate the wear rate of individual inserts while considering the overall durability of the tool, making it easier to obtain data on tool wear and chip formation. (2) The symmetric installation of three inserts ensures better cutting stability during high-speed milling, reducing the vibrations and noise caused by imbalance, thereby improving the cutting stability and accuracy. (3) Symmetrically installed inserts facilitate easier installation, adjustment, replacement, and maintenance during the experiment.

To improve the accessibility of the current tool wear status information across multiple passes, the workpiece is machined as a rectangular body measuring 300 × 300 × 2000 mm. The insert details are shown in Table 3. The process of dry cutting is employed, and the cutting parameters are established based on the actual machining and cutting of the water-chamber head, which closely resemble actual machining scenarios. The specifications are as follows: 195 rpm for the spindle speed, 600 mm/min for the feed rate, 1 mm for the cutting depth, and 150 mm for the milling width.

**Figure 1:**
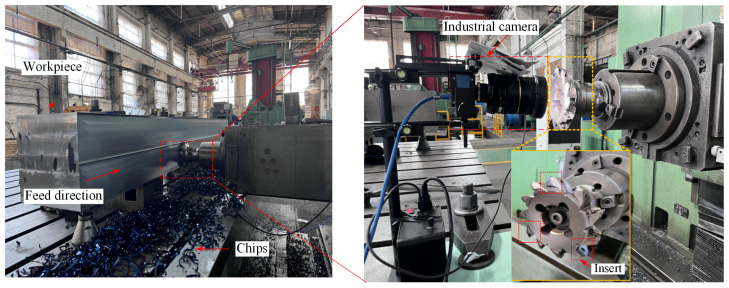
Milling experiment site.

In order to precisely and effectively assess the degree of tool degradation, it is essential to pre-set the spatial alignment and optical distance between the industrial camera and the insert beforehand. This setup involves milling a complete plane each time, with the cutter moving a distance of 4000 mm. During this process, the milling machine table needs to rotate counterclockwise by 90 degrees, and the milling cutter disk must rotate at a precise angle. These adjustments are vital for the precise determination of the tool wear value. The tool wear is measured using an industrial camera MARS-1230-23U3C of Daheng in Beijing, China, and the milling experiment site is depicted in Figure 1.

### 2.3. Chip Data Collection

**Figure 2:**
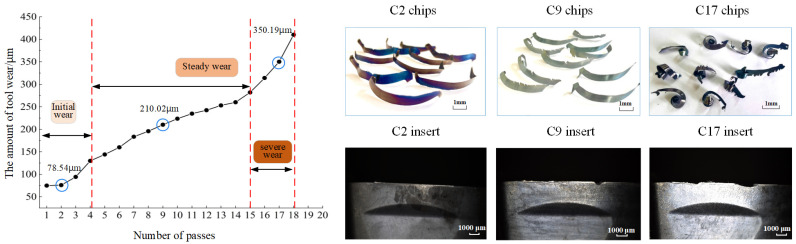
Chip morphology and tool wear under different passes.

The products of the tool and workpiece coming into direct contact are chips, and the color of the chips is directly related to the temperature at which the chip is being cut, and the color of chips is affected by the state of tool wear as well as the cut-in and cut-out of the tool. For every 4000 mm length of cutting, 90 chips from the intermediate milling process are gathered in order to collect the chip data that can describe the tool wear conditions. There are 18 experiments in total, and the 18 passes are numbered C1–C18, and the chips obtained from the ith pass are defined as Ci chips. The 18 passes represent the initial tool wear stage (C1–C3), steady tool wear (C4–C15), and severe wear stage (C16–C18). Figure 2 illustrates the trends in tool wear and chip morphology at various tool wear stages in the 18 groups. According to the international ISO 8688-2 standard [19], tool wear is measured by the average wear on the flank face of all cutting edges. Specifically, the wear amount (VB value) on the flank face of the cutting inserts is measured. Considering the practical ease of measurement, this paper quantitatively assesses the degree of tool wear by averaging the wear amounts on the flank faces of multiple cutting inserts.

Significant color differences in the chips produced at various tool wear stages are revealed by analyzing Figure 2. Initially, the chips exhibit a range of colors, including yellowish-brown, purple, red, and blue, with some chips showing a blend of multiple shades. As the tool progresses to the steady wear stage, the primary chip color changes to blue, albeit with varying intensities. The chip color eventually darkens to deep purple and deep crimson throughout the severe wear stage. Throughout the milling process of 508III steel, the chip color transitions in the following sequence: yellowish-brown → brownish → purple → dark purple/dark blue → light blue → blue-green → purple-black. An important indicator of the approaching severe wear phase is when a significant portion of the back of the chip shifts to light blue. Therefore, monitoring changes in chip color serves as a critical marker of distinct tool wear stages.

## 3. Chip Morphology Analysis

**Figure 3:**
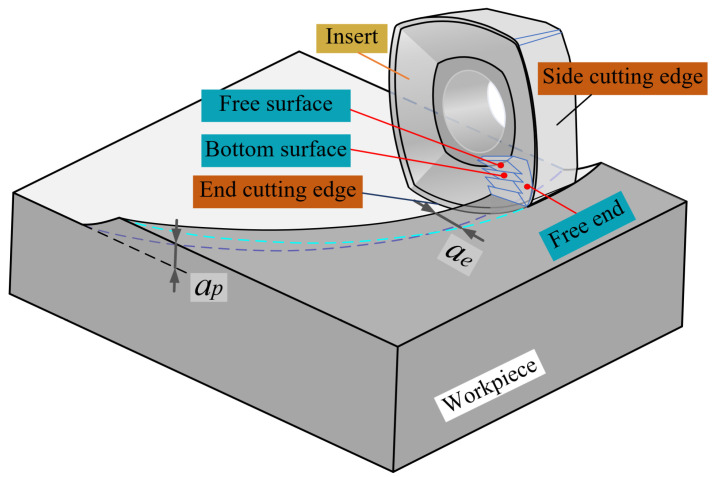
Three-dimensional schematic diagram of chips formation.

To distinctly describe the contact between the tool and the workpiece, as well as the complex geometry of the insert when actually making the cut, the three-dimensional observation of the chip formation process is also crucial in understanding the formation of machined surfaces and the chip formation condition at the time of machining. As shown in Figure 3, a three-dimensional schematic of the chip formation process characterizes the motion relationship between the insert and the cutting layer of metal, and contains cutting parameters including the radial depth of cut *a_e_* and the axial depth of cut *a_p_*. Meanwhile, the chip morphology is characterized from multiple perspectives during the milling process, defining the chip free surface, chip bottom surface, chip free end, and chip non-free end.

In order to characterize the chip micro-morphology, Kroll reagent is used to etch the polished chips, and the morphology of different surfaces of the chips was monitored by using super depth-of-field microscopy and scanning electron microscopy (SEM) at different numbers of tool strokes. Changes in the elemental composition of the chip surface were analyzed by X-ray energy-dispersive spectroscopy (EDS).

### 3.1. Chip Free Surface Morphology Analysis

Due to the limitations of the actual processing conditions and part size, the actual cutting parameters of heavy milling 508III steel generally include lower cutting speeds, larger cutting depths, and the formation of banded chips. The free surface of the chip is shaggy, forming a shallow depth, small spacing, and the free end of the wider, non-free end of the narrower shear slip deformation band; in the shear slip deformation band, the shear deformation is dense and small.

**Figure 4:**
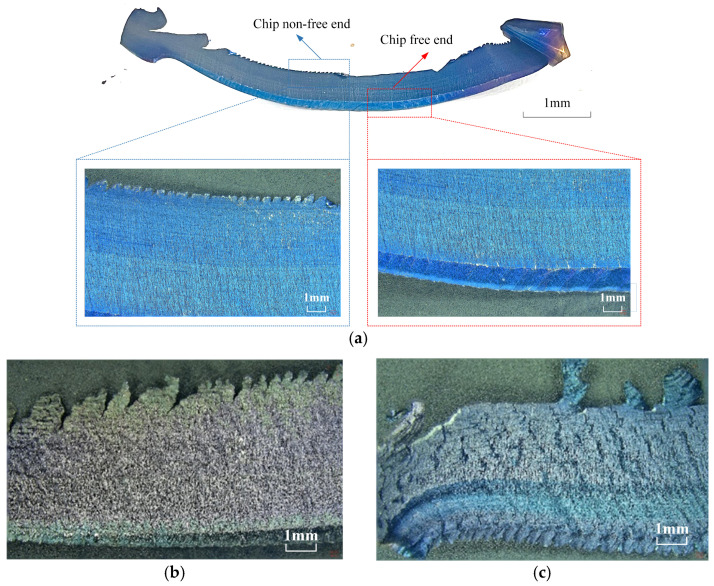
Free surface morphology of chips with different number of passes. (**a**) Free surface morphology of the chip at the second pass. (**b**) Free surface morphology of the chip at the 8th pass. (**c**) Free surface morphology of the chip at the 18th pass.

Figure 4 shows the macroscopic morphology of the free surface of the chip under different numbers of tool strokes (magnified 20×). Comparing the free surface morphology under different numbers of tool strokes, it can be claimed that in the initial wear stage of the tool (under the 3rd pass), the free surface of the chip is relatively fine; the shear deformation slip bands are dense and uniform; with the increase in tool wear (under the 8th pass), the free surface of the chip becomes rough, and the shear deformation slip bands are not obvious; and when the tool wear is more severe (under the 18th pass), the free surface of the chip is obviously cracked, and crack structures appear parallel to the chip width. When the tool wear is more severe (18th pass), the free surface of the chip produces an obvious cracking phenomenon, and the crack structure parallel to the width of the chip appears. The chip’s color is obviously different under a different number of passes, and the color of the chip varies along the chip width direction. The chip’s free surface appears blue under the 3rd pass, dark purple under the 8th pass of tool walking, and blue-green under the 18th pass of tool walking and there is a clear strip to exhibit the color band.

**Figure 5:**
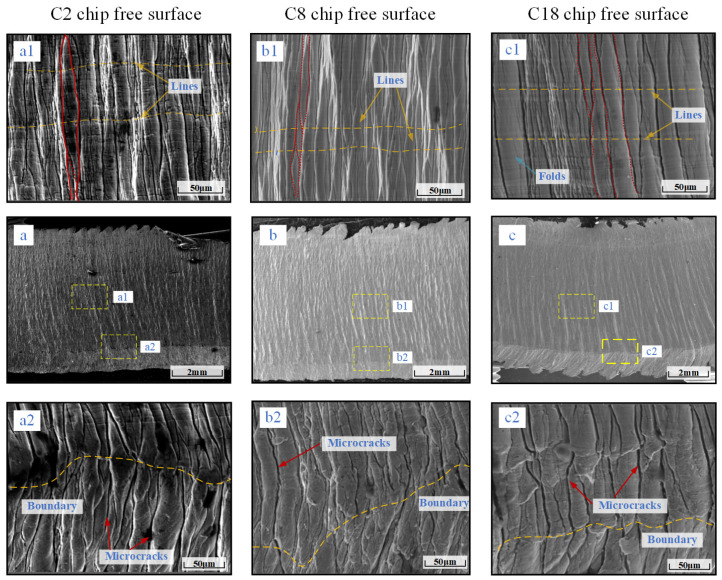
SEM image of the free surface of the chip with different numbers of tool strokes. (**a**–**c**) Free surface of C2, C8 and C18 chips. (**a1**–**c1**) A larger view of upper free surface of C2, C8 and C18 chips. (**a2**–**c2**) A larger view of lower end of free surface of C2, C8 and C18 chips.

In order to further investigate the free surface characteristics of chips and reveal the free surface micro-morphology of chips under different tool wear conditions, SEM is utilized to examine the free surfaces of chips under different numbers of tool strokes, as shown in Figure 5.

In heavy milling of 508III steel, the chip free surface shear-slip with a substantial amount of folds are seen, and the chip free surface is characterized by non-periodic “pomelo meat-shaped” and “layered-rock shaped” characteristics [20]. As shown in the red areas (a1), (b1) and (c1) in Figure 5. In Figure 5, the tool’s tip region is depicted as having a “pomelo meat-shaped” free surface. This indicates that the tool is in its initial and usual wear stages, as indicated by the number of passes being C2 and C8. Chip formation process occurred in the unstable high plastic flow, and accompanied by folding deformation, the formation of “pomelo meat-shaped” folds. Numerous researchers have identified comparable phenomena when investigating the machining of plastic materials, including copper and tantalum. The deformation of chips is unstable when cutting plastic metals, resulting in unequal redundancy deformations and stacking, resulting in a sinuous flow [21,22]. Due to the sinuous flow, severe plastic deformation during the cutting process leads to chip accumulation on the front face, which is the primary cause of the development of “pomelo meat-shaped” chips.

The microcracks that form between the fold units and the “pomelo meat-shaped” folds are substantially inhibited when the tool is in the severe wear phase with the C18 pass. The free surface of the chip tends to be “layered rock-shaped”. The work material hardens, increasing the chip’s hardness, brittleness, and bending, which prevents plastic flow and causes further microcracks to form. The chip becomes “layered rock-shaped” instead of “pomelo meat-shaped”. In Figure 5(a1,b1,c1), which compare the free surfaces of the chips, the chip flow lines are parallel with one another. As the tool wear increases, the lines deepen, which is related to the chip flow through the front face. There is a certain relationship with the front face of the contact effect. In the comparison of Figure 5(a2,b2,c2), it can be seen that the free surface of the chip and the free end of the chip form an obvious boundary. In the boundary area, the C2 and C8 chip free-surface organization is denser, there is a shallow microcrack, the tool wear stage of C18 chips produces deep cracks, and the chip free ends of the nearby areas exhibit “pomelo meat-shaped” characteristics.

### 3.2. Chip Bottom Surface Morphology Analysis

During the milling process, similar to the process for other cutting methods, the chips are guided toward the front section of the tool. The front face of the tool and the underside of the chip approach each other, limiting the plastic deformation of the chip’s lower surface by the tool’s front face. Therefore, when the chip slides on the tool’s front face, the chip’s bottom surface carries high contact pressure and friction. A smooth chip base is formed at the tool–chip contact interface after plastic flow deformation caused by friction and high contact stress. During the cutting process, the bottom surface of the chip is in close contact with the front surface of the tool, which limits the contact of oxygen in the air with the chip and reduces the degree of oxidation, so the color is deeper than on the free surface of the chip.

**Figure 6:**
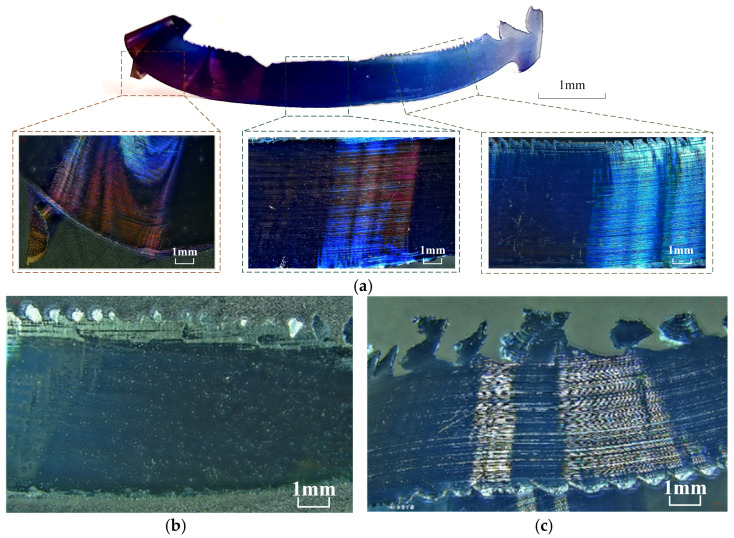
Bottom surface morphology of chips under different numbers of passes. (**a**) Bottom surface of the chip under the second pass. (**b**) Bottom surface of the chip under the 8th pass. (**c**) Bottom surface of the chip under the 18th pass.

Figure 6 shows the morphology of the chip bottom surface under different numbers of tool strokes (magnified 20×). Although the distribution of contact stress, friction and temperature at the tool–chip interface varies somewhat during various phases of tool wear, the chip bottom surface has very similar characteristics. In contrast, the stripes formed on the chip bottom surface along the chip flow direction are caused by insert wear, microchipping, and hard spots, but are smooth overall. As illustrated in Figure 6a, in the early stage of tool wear, as the new insert enters the cutting area, the tool–workpiece contact friction and tool–chip contact friction are small, the cutting temperature is low, the chips take away most of the heat of cutting, and the chips have incomplete contact with the tool. At this time, the bottom surface of the chip shows a variety of colors, and these characterize the temperature change in the fracture formation process of each chip. As shown in Figure 6b, at the 8th pass, the tool is in the steady-wear stage, the chip is smooth, and the parallel stripes are not obvious. As shown in Figure 6c for the 18th pass, the tool is in the stage of severe wear; this time, the tool wear is more serious, and the bottom surface of the chip produces more obvious parallel stripes and produces a more serious scoring phenomenon, indicating that at this time, the front face of the tool produces crescent puddle wear, groove wear, breakage, or the existence of a potential hard point when the chip flows through the front face, producing intense wear and a scoring phenomenon.

**Figure 7:**
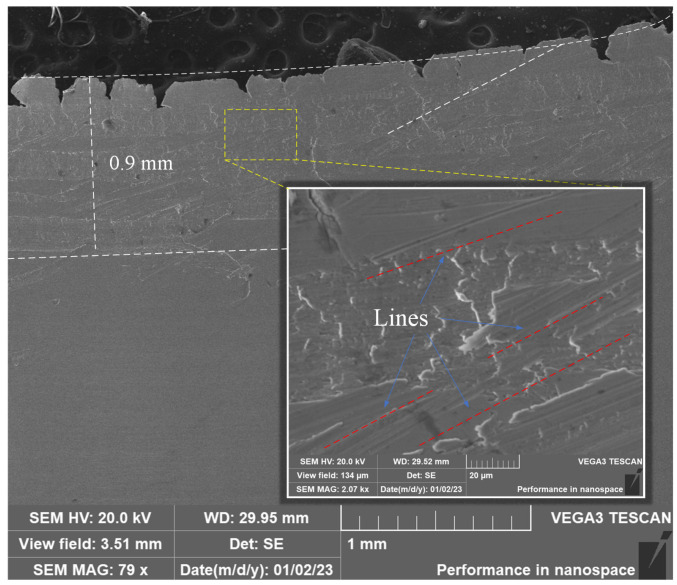
Microscopic morphology of the bottom surface of the chip under the 6th pass.

**Figure 8:**
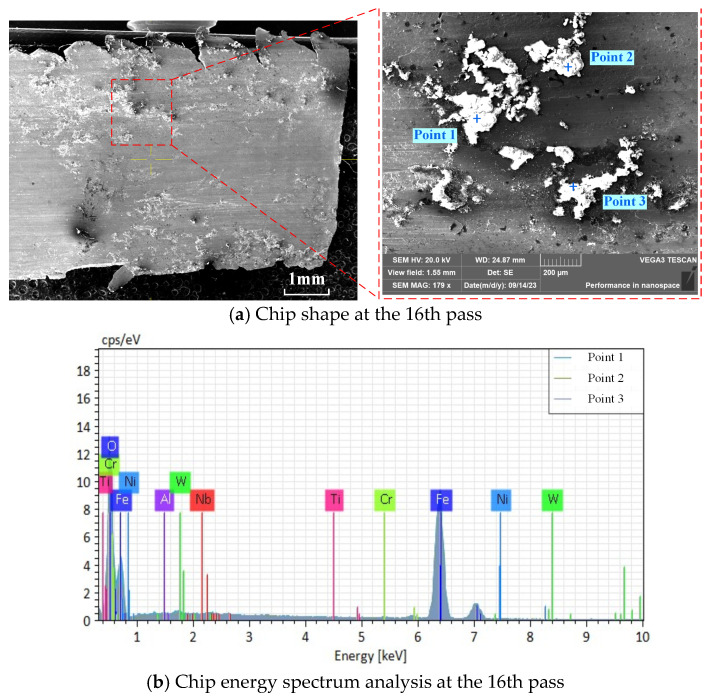
Chip morphology and energy spectrum analysis at the 16th pass.

In order to reveal the contact characteristics between the front face of the tool and the chips, SEM is used to examine the bottom surface of the chips collected at different numbers of tool passes. The X-ray EDS is used to analyze the changes in the chemical composition of the chip surface. The microscopic morphology of the bottom surface of the chips under the sixth tool pass is shown in Figure 7. The figure illustrates that about 0.9 mm from the free end of the chip, there is distribution of river-like grain, while the other areas of the surface are relatively smooth, with a parallel grain angle of about 22°; it can be deduced that the chip along the front surface of the chip outflow, close to the non-free end of the chip and the front surface of the tool friction, is greater, resulting in the 22° scratch-angle phenomenon. Figure 8 shows the chip morphology and energy spectrum analysis under the 16th tool pass, and Table 4 shows the normalized mass percentage of the elements of the chip energy-spectrum analysis. As the degree of tool wear becomes larger, the bottom surface of the chip appears to be bonded and black particles can be seen, indicating that at this time the cutting temperature is higher and the tool and chip produce intense contact and friction, resulting in bonding. The white bond on the bottom surface of the chips contains elements such as Fe, Cr, Ni, Nb, Al, Ti, O, W, etc. After the energy spectrum analysis, the chips contain elements such as Fe, Cr, Ni, Nb, Al, Ti, O, W, etc., and Al, Ti, and W are the elements of the insert coating. In the process of the chip flowing out of the cutting tool on the front face of the cutting tool, with the aggravation of the tool abrasion, the chip produces sharp friction with the tool and the cutting tool, so that the cutting temperature rises, and the chip produces a bond with the cutting tool. The oxygen content of the bonded material is high, and it can be concluded that the bonded material is an oxide; at this time, an oxidation reaction occurs between the insert and the chip.

**Figure 9:**
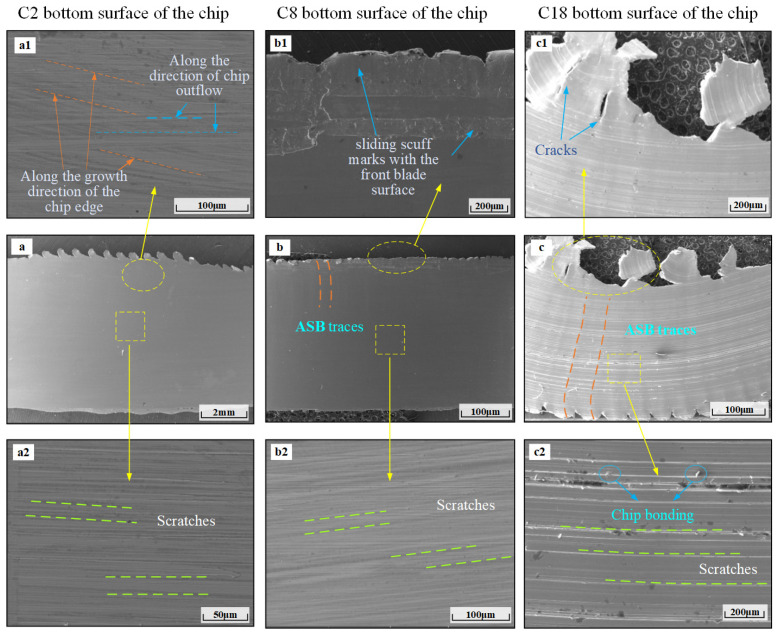
SEM images of chip bottom surface at different tool wear stages. (**a**–**c**) Bottom surface of C2, C8 and C18 chips. (**a1**–**c1**) A larger view of bottom surface of upper in C2, C8 and C18 chips. (**a2**–**c2**) A larger view of bottom surface of lower end in C2, C8 and C18 chips.

In order to analyze the change rule of the chip bottom surface at different wear stages, Figure 9 shows the SEM images of the chip bottom surface at different tool wear stages. By observing Figure 9a–c, it can be concluded that severe wear results in more obvious adiabatic shear band traces on the bottom surface of the chip than the initial wear and steady-wear stages (at the mark ASB in Figure 9), through the local magnification of the non-free end edge of the bottom surface of the chip in the three stages of the tool wear. It can be seen that the bottom surface of the chip in the initial wear stage is relatively smooth, with no obvious adiabatic shear traces and no changes in the distance from the non-free end of the edges. When the chip surface is magnified 815×, it can be seen that there are slip traces along the direction of chip edge growth. Evidently, there are traces of slippage along the growth direction of the chip edge and also along the direction of chip outflow. In Figure 9(b1), it is observed that the non-free end chip edge produces sliding scuff marks with the front insert surface, and the chip edge size is smaller in the initial wear and steady-wear stages. Based on Figure 9(c1), it can be concluded that the severe wear stage produces deeper cracks and a larger chip edge size at the edge of the chip and cracks and scratches on the chip edge, and the bottom surface of the chip is rougher in the sharp-wear stage compared to the initial wear and steady-wear stages. The middle part of the chip surface in the three phases of wear on a tool is enlarged locally, and it can be concluded from Figure 9(a2,b2,c2) that the acute wear stage has more serious chip bonding phenomenon and parallel scratches. The scratches are wide and deep, and the chip surface in the initial wear stage is relatively smooth. Scratches are generated due to the chip cutting through the front face, and the chip bottom surface is subjected to greater contact pressure and friction, irregularity of the cutting edge, impurities in the material or debris accumulation in the vicinity of the tool, and the tool passes across the chip bottom surface to produce scratches. Severe wear-stage scratches are the most serious; this is due to the tool wear phenomenon being more serious, so that the knife–chip friction becomes greater, meaning that in the sharp-wear stage, the tool chip bottom surface scratches are deep and the bottom surface is rougher.

**Figure 10:**
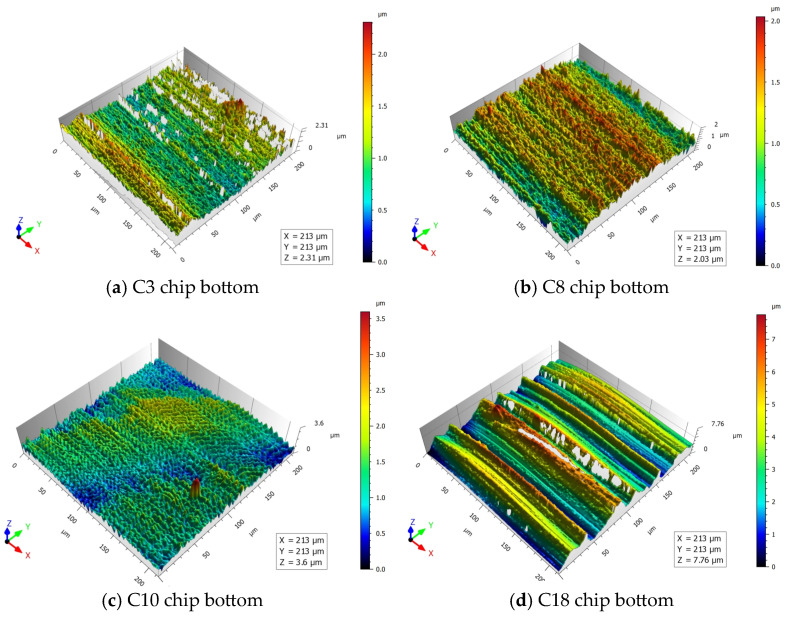
Chip bottom surface morphology.

The bottom surface of the chip generates intense friction with the front face of the tool. In order to further investigate the relationship between the traces left on the bottom surface of the chip by the flow of chips across the front face of the tool and the tool wear, the three-dimensional surface texture parameters of the bottom surface of the chip were assessed utilizing a CCI Map Talor Hobson non-contact white-light interferometer. In this paper, the numerical value measured by the Z-axis of the white light interferometer is used to characterize the roughness R_Z_ value of the chip bottom surface. The surface morphologies of the bottom surface of the chips exhibiting various degrees of tool wear are shown in Figure 10.

As illustrated in Figure 10, the tool wear increases with the rise in cutting times. It is evident that the roughness of the bottom surface of the chip becomes larger; when the tool is in the initial wear stage, the roughness of the bottom surface of the chip is about 2 μm, with the progression of tool wear, and when the tool wear enters the late stage of the steady-wear stage, the roughness of the bottom surface of the chip increases to 3.6 μm. When the tool is about to reach the standard of bluntness, the tool enters the stage of severe wear. The roughness of the bottom surface of the chip is 7.76 μm. The tool comes into direct contact with the front face of the tool, and the alteration in the morphology of the chip’s bottom surface can indicate the level of contact between the chip and the tool. Additionally, tool wear is the primary factor leading to scratches at the bottom of the chip. The primary cause of chip curl is the friction between the chip and the front tool surface, and the increase in the roughness of the chip bottom surface intensifies the extrusion between the chip and the front tool surface so that there are different residual strains in the direction of the chip thickness, and the chip grains are flipped, causing the chip to curl.

## 4. Analysis of Chip Edges Morphology

As tool wear rises during the chip creation process, the morphology of the chips can provide certain insights into the actual cutting process. This reflection is influenced by extrusion pressure, friction, heat, chemical reactions, and other significant factors. In addition to the plastic flow along the front face of the chip (free surface and bottom surface), there will be a certain amount of plastic deformation in the direction of the chip width, i.e., lateral plastic flow, which is reflected in the free end of the chip, and the direction of the non-free end is also different. Therefore, to further explore the chip morphology characteristics, it is necessary to reveal the different wear conditions of the tool chip lateral plastic flow process in the chip edge pattern change rule.

Cutting difficult-to-machine materials often takes place in high-temperature conditions, and in this case, the workpiece material by the extrusion of the tool edge will flow to the free end, resulting in extrusion deformation of the nearby material. This phenomenon is observed on the plastic side of the flow of metal materials, including the machined surface of the lateral plastic flow and the side of the plastic flow of the chip. Due to the lateral plastic flow, metal residues and cutting edges will be left on the machined surface, and the chip shape will produce chip edges and changes in chip width.

When the chip flows through the front face, the chip is subjected to cutting layer extrusion and additional pressure and curl from the next chip unit, as the chip removal rate is much lower than the tip feed rate. Due to the poor thermal conductivity of the material, the instantaneous cutting heat generated during heavy milling is large, resulting in viscosity of the high-temperature material, as well as a strong frictional resistance to chip flow out of the front side, which leads to resistance to the plastic flow of chips along the front side. The principle of the law of least resistance [23] posits that metal materials have a tendency to deform in the direction of least resistance, leading to non-uniform plastic deformation across the chip’s width. As a result, the chip’s width increases beyond its initial dimensions upon detachment from the workpiece, thereby playing a role in the formation of chip edges.

**Figure 11:**
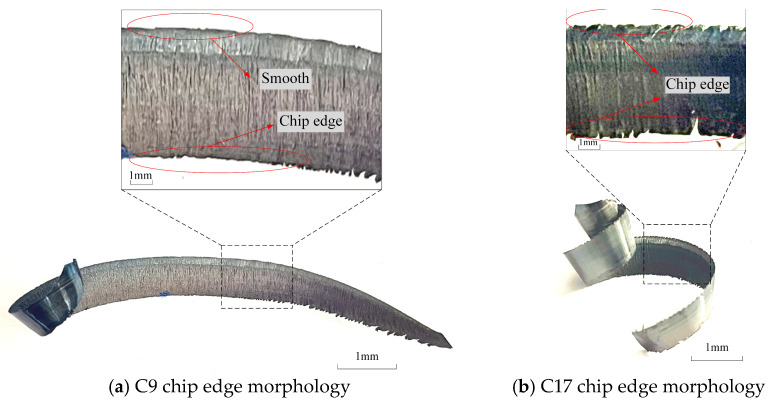
Chip edge profile under different number of passes.

The workpiece material undergoes a brittle-plastic transformation at high speeds and high strain rates and chips are formed instantaneously. Unlike in conventional cutting, the experiment found that during the initial tool-wear stage, the non-free end of milling 508III steel chips showed different degrees of burr patterns, while the free end was smoother. When the tool wear reaches a certain level, the chip edge on the free end of the chips forms gradually during the 11th pass in heavy milling of 508III steel, as observed from the chip non-free end and the free end of the emergence of the chip edge morphology, and there is a more obvious change, as shown in Figure 11.

**Figure 12:**
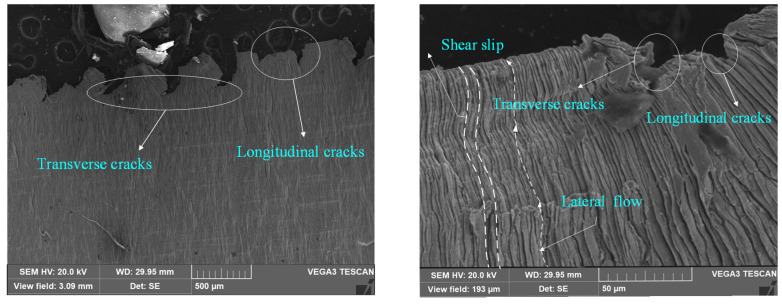
C6 chip non-free end chip edges.

The examination of chip morphology under various tool wear conditions, as depicted in Figure 12, indicated the presence of lateral plastic deformation and significant cracks. The chips exhibited both transverse and longitudinal cracks, particularly near the chip edges. Longitudinal cracks were observed to be perpendicular to the adiabatic shear zone, while transverse cracks were aligned parallel to the two scale structures, as depicted in Figure 12. The microcracks on the chips belong to the fracture mechanics behavior of the material, and its generation indicates that there is damage and gradual evolution of the cutting layer metal during the cutting process, and the cracks will intermittently scratch the cutting tool’s front face and cutting edge, causing high-frequency vibration of the cutting system and affecting the surface quality of the workpiece.

## 5. Analysis of Chip Curl Pattern in Heavy Milling 508III Steel

**Figure 13:**
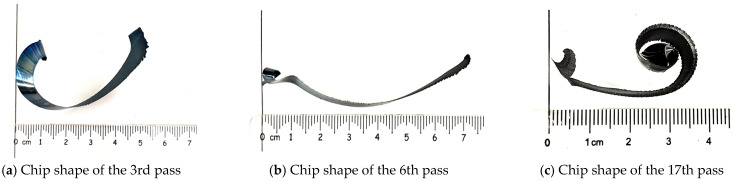
Chip curl pattern with partial number of passes.

During the experiments conducted for milling 508III steel, it was found that even under the same cutting parameters, the chips produce different curl patterns with increasing tool wear. Figure 13 shows the chip curl morphology under heavy milling 508III steel with a partial number of tool strokes.

From Figure 13, it can be seen that during the initial wear stage, the chip morphology shows C-shaped chips with different curvature. This particular type of chip is produced when there is a shift in the direction of the chip force at the onset of the cutting process, resulting in heightened wear of the sharp edge due to stress concentration. The interplay between cutting resistance on the shear face and force on the frontal face generates a bending moment that notably alters the shape of the chip. During the steady-wear stage of the tool, the chip shape becomes a strip chip, the chip becomes longer, the radius of curvature becomes larger, the chip grows first to produce a lateral curling tendency, and then becomes an upward curling strip chip. When the tool is in the stage of severe wear, the degree of upward curling of the chip becomes larger, and the radius of curvature of the end of the chip gradually becomes smaller.

## 6. Conclusions

This research centered on the chips generated during the milling of the difficult-to-machine material 508III steel. An empirical investigation was carried out on the heavy milling of 508III steel at the operational machining site to explore the macroscopic and microscopic characteristics of the chips under different tool wear conditions. Additionally, the study scrutinized the morphology of chip edges, identified their sources, and assessed the curling structure of the chips produced during heavy milling of 508III steel. The conclusions of the study are as follows:

(1) The chips produced during heavy milling of 508III steel differ significantly from ordinary chips, and there are notable differences in the macro-morphologies and micro-morphologies of the chip’s free surface at different stages of tool wear. Macroscopically, the size of the chips is large and the color of their free surfaces varies significantly; the color of the chip varies along the chip width direction. When the tool wear reaches the stage of severe wear, there is a clear strip of color band on the free surface of the chip. Microscopically, there are a lot of folds on the free surface of the chip with shear slip, and the free surface of the chip is characterized by a “pomelo meat” shape in the initial and steady wear stages of the tool. During the severe wear stage, the “pomelo meat-shaped” folds become relatively suppressed, and microcracks start to appear between the fold units. Additionally, the free surface of the chip tends to be close to the “layered-rock shape”.
(2) Due to the action with the front insert surface, a more obvious scratch is formed on the bottom surface of the chip. As the tool wear increases, the scratching and plowing phenomenon is more significant. The initial wear and steady wear stage of the chip edge size is small. Severe wear on the chip edge produces deeper cracks and a larger chip edge size. Furthermore, all the cracks and scratches are on the chip edge. The bottom surface of the chip under the severe wear stage has more obvious adiabatic shear band marks than the initial wear and steady-wear stages. The severe wear stage has more serious chip bonding phenomena and parallel scratches, and when the tool is about to reach the dull standard, the roughness of the chip bottom surface reaches 7.76 μm.
(3) The non-free and free ends of chips show different patterns at different wear stages. In the initial stage of tool wear, the non-free end of milling 508III steel chips shows different degrees of chip edge morphology, while the free end is relatively smooth. The chip edge on the free end of the chips formed gradually during the 11th pass in heavy milling of 508III steel; both the non-free end and free end of the chip show chip edge morphology, and there are more obvious changes. During the examination of chip morphology at various stages of tool wear, it was noted that the chips not only exhibit lateral plastic flow phenomena but also manifest noticeable cracks.
(4) Different chip patterns at different wear stages. During the initial phase of tool wear, the chip exhibits varying degrees of C-type curling. As the tool progresses to the steady-wear stage, the morphology of the chip transforms into a strip configuration characterized by elongation and a greater radius of curvature. The expansion of the chip initiates with lateral curling, eventually evolving into an upward curling pattern that is typically seen in strip chips. During the severe wear stage of the tool, there is an increase in the degree of upward curling of the chip, leading to a gradual reduction in the radius of curvature at the end of the chip.

## Figures and Tables

**Table 1:** The elemental makeup of 508III steel (%).

Ingredient	w (C)	w (Si)	w (Mn)	w (P)	w (S)	w (Cr)	w (Ni)	w (Mo)	w (V)
Norm	0.17~0.23	0.15~0.30	1.12~1.50	≤0.012	≤0.015	≤0.25	0.57~0.93	0.40~0.60	≤0.01
Real measurement	0.18	0.17	1.4	0.005	0.003	0.14	0.79	0.51	0.005

**Table 2:** Mechanical properties of 508III steel.

Temperature	$\sigma_{0.2}/$Mpa	$\sigma_{b}/$Mpa	$\Delta_{s}/$%	Ψ/%
Longitudinal room temperature	530	632	27.5	75
Longitudinal 350 °C	415	563	19.5	75
Lateral room temperature	481	614	29	75
Lateral 350 °C	481.5	555.5	19.5	75

**Table 3:** Insert-related parameters.

Relevant Parameters	Corner Radius	Cutting Edge Length	Insert Thickness	Coating Name	Coating Thickness
Values	1.5 mm	19 mm	6.35 mm	CTPP235	4 μm

**Table 4:** Normalized mass ratio (%) of bonded material on chip surface.

Points	O	Al	Ti	Cr	Fe	Ni	Nb	W
Point 1	42.89	0.06	0.00	0.20	56.64	0.07	0.12	0.02
Point 2	39.22	0.00	0.15	0.02	60.51	0.10	0.00	0.00
Point 3	38.35	0.45	0.14	0.06	60.47	0.52	0.02	0.00

## Data Availability

The original contributions presented in the study are included in the article, further inquiries can be directed to the corresponding author.

## References

[1] Liu X., Li X., Ding M., Yue C., Wang L., Liang Y., Zhang B. (2021). Intelligent Management and Control Technology of Cutting Tool Life-cycle for Intelligent Manufacturing. J. Mech. Eng..

[2] Rai R., Tiwari M., Ivanov D., Dolgui A. (2021). Machine learning in manufacturing and industry 4.0 applications. Int. J. Prod. Res..

[3] Mikhailov V., Kovelenov Y., Bolotskikh V. (2018). Simulation of Chip Formation and Fracture in the Design of Complex Turning Inserts. Russ. Eng. Res..

[4] Khashaba U. (2024). Analysis of surface roughness, temperature, short aging, and residual notched and bearing strengths in supported drilling of thin GFRP composites. Alex. Eng. J..

[5] Rabinarayan B., Amlana P., Kumar A. (2023). Machinability characteristics analysis of hard turning operation on AISI 4340 steel using physical vapor deposition multilayer coated carbide cutting tool in the dry environment. Proc. Inst. Mech. Eng. Part E J. Process Mech. Eng..

[6] Cheng Y., Gai X., Guan R. (2023). Temperature Experiment and Parameter Optimization of Cemented Carbide Tool in Milling 508III Steel. Materials.

[7] Cheng Y., Li C., Yuan Q., Lv Q., Liu L. (2019). Experiment and model of cutting force of heavy-duty milling water chamber head material. SN Appl. Sci..

[8] Wu M., Zhao X., Chen Y., Xu M., Cheng Y., Li L. (2017). Reaserch on Mechanism and Experimental of Chip Breaking during High Pressure Cooling Turning of Superalloys with PCBN Tool. J. Mech. Eng..

[9] Devotta A., Beno T., Löf R. (2017). Finite element modelling and characterisation of chip curl in nose turning process. Int. J. Mach. Mach. Mater..

[10] Gaurav S. (2024). Experimental investigation of micro-pillar textured WC inserts during turning of Ti6Al4V under various cutting ffuid strategies. J. Manuf. Process..

[11] Sun S., Brandt M., Dargusch M. (2017). Effect of tool wear on chip formation during dry machining of Ti-6Al-4V alloy, part 1: Effect of gradual tool wear evolution. Proc. Inst. Mech. Eng. Part B J. Eng. Manuf..

[12] Vincent W., Maher B., Gilles D. (2015). The relationship between the cutting speed, tool wear, and chip formation during Ti-5553 dry cutting. Int. J. Adv. Manuf. Tech..

[13] Cui X., Zhao B., Jiao F., Zheng J. (2015). Chip formation and its effects on cutting force, tool temperature, tool stress, and cutting edge wear in high- and ultra-high-speed milling. Int. J. Adv. Manuf. Tech..

[14] Demirpolat H., Binali R., Patange A. (2023). Comparison of Tool Wear, Surface Roughness, Cutting Forces, Tool Tip Temperature, and Chip Shape during Sustainable Turning of Bearing Steel. Materials.

[15] Gdula M., Mrowka-Nowotnik G. (2023). Analysis of tool wear, chip and machined surface morphology in multi-axis milling process of Ni-based superalloy using the torus milling cutter. Wear.

[16] Das A., Bajpai V. (2023). Machinability analysis of lead free brass in high speed micro turning using minimum quantity lubrication. Cirp. J. Manuf. Sci. Tec..

[17] Che J., Zhang Y., Wang H., Chen J., Du M. (2023). An experimental and numerical study on the wear mechanism of cutters on workover bits under thermo-mechanical coupling. Geoenergy Sci. Eng..

[18] Guan R., Cheng Y., Zhou S., Gai X., Lu M., Xue J. (2024). Research on tool wear classification of milling 508III steel based on chip spectrum feature. Int. J. Adv. Manuf. Tech..

[19] Cheng Y., Lu M., Gai X., Guan R., Zhou S., Xue J. (2024). Research on Multi-Signal Milling Tool Wear Prediction Method Based on GAF-ResNext. Robot CIM-Int. Manuf..

[20] Wang R., Wang X., Pei Y. (2023). The effects of cryogenic cooling on tool wear and chip morphology in turning of tantalum-tungsten alloys Ta-2.5W. J. Manuf. Process..

[21] Anirban M., Ho Y., Yang G. (2017). Sinuous flow and folding in metals: Implications for delamination wear and surface phenomena in sliding and cutting. Wear..

[22] Udupa A., Sugihara T., Mann J. (2019). Glues make gummy metals easy to cut. J. Manuf. Sci. Eng..

[23] Cui F., Xie Y., Dong X., Hou L. (2014). Simulation analysis of metal flow in high-speed cold roll-beating. Appl. Mech. Mater..

